# Plant Growth-Promoting Rhizobacteria Enhance Sweet Cherry Root System Development Through the Production of Volatile Organic Compounds

**DOI:** 10.3390/foods14132369

**Published:** 2025-07-03

**Authors:** Nan Zeng, Rutao Gai, Dandan Wang, Jiahe Pang, Dingcun Zhang, Junliang Ge, Xinyue Bi, Zhiyong Zhang, Ning Zhang, Bingxue Li

**Affiliations:** 1 College of Land and Environment, Shenyang Agricultural University, Shenyang 110866, China; zengnan1015@163.com (N.Z.); 13274258130@163.com (R.G.); xiaoguanjia98@163.com (D.W.); pangjiahe2023@163.com (J.P.); xinyuebi2023@syau.edu.cn (X.B.); zhangzy@syau.edu.cn (Z.Z.); 2 College of Bioscience and Biotechnology, Shenyang Agricultural University, Shenyang 110866, China; zdc191817@163.com (D.Z.); m18342862570@163.com (J.G.)

**Keywords:** cherry, PGPR, VOCs, *Pantoea ananatis*, *Burkholderia*, growth-promoting, microbial community

## Abstract

Sweet cherry (*Prunus avium* L.), as a high-economic-value fruit with both nutritional and health functions, faces severely constrained plant growth due to underdeveloped root systems and suboptimal orchard site conditions. Plant growth-promoting rhizobacteria (PGPR) demonstrate application potential in regulating plant development and improving soil structure through the release of volatile organic compounds (VOCs). This study systematically evaluated the effects of VOCs from three PGPR strains—*Pantoea ananatis* D1-28, *Burkholderia* sp. D4-24, and *Burkholderia territorii* D4-36—on cherry root development and rhizosphere microbial communities. The results indicate that when D1-28 and D4-24 strains were at 10^3^ cfu·mL^−1^ and D4-36 was at 10^5^ CFU·mL^−1^, their VOCs exhibited optimal growth-promoting effects. Compared with the control group, significant improvements were observed in cherry seedling parameters, including plant height, total biomass, root length, root surface area, and root volume. The VOCs from these strains synergistically promoted plant growth by regulating auxin synthesis pathways in cherry roots while enhancing the relative abundance of beneficial rhizosphere microorganisms. This study establishes the strain-concentration–effect relationship, providing a theoretical foundation to optimize soil microbial environments and promote cherry root development using PGPR.

## 1. Introduction

Sweet cherry (*Prunus avium* L.), as a high-economic-value fruit, is rich in anthocyanins, vitamin C, and antioxidants, combining nutritional and health-promoting functions, leading to a continuous growth in market demand [1,2]. However, most of the sweet cherry orchards in China are characterized by poor site conditions and shallow soil layers [3,4]. Furthermore, cherry trees have weak root system development, a limited root distribution range, and low absorption capacity, making them highly sensitive to changes in soil environmental conditions [5]. These unfavorable factors severely restrict the growth and development of cherry trees. Traditional solutions rely on chemical inputs, but long-term use exacerbates soil degradation and increases the risk of heavy metal residues in fruit, which is contrary to consumers′ expectations for green food [6,7,8,9,10]. Therefore, there is an urgent need to find an economically viable, efficient, and environmentally friendly method to improve cherry root function, promote plant growth and development, and ensure the sustainable development of the sweet cherry industry.

Plant growth-promoting rhizobacteria (PGPR) have garnered significant attention in the agricultural field as beneficial microorganisms that enhance plant growth and stress resistance [11,12,13]. Through mechanisms such as nitrogen fixation, phosphate solubilization, and the production of phytohormones, PGPR directly or indirectly promote plant growth [14,15]. Notably, certain PGPR strains produce volatile organic compounds (VOCs), which demonstrate substantial potential in regulating plant growth and development, defending against pests and diseases, and improving soil structure [16,17,18,19,20]. Compared with conventional chemical fertilizers and pesticides, utilizing PGPR and their VOCs to stimulate cherry seedling growth offers natural, eco-friendly, and sustainable advantages, providing new perspectives and approaches for the development of green agriculture [21,22,23].

Despite preliminary explorations of the growth-promoting mechanisms of PGPR-VOCs in model crops such as tomato and *Arabidopsis* [24,25,26], significant research gaps remain in their application to woody fruit trees (particularly cherry): (1) the complex root architecture and extended growth cycles of cherry trees may lead to interaction mechanisms with VOCs that substantially differ from those in herbaceous plants; (2) the concentration effects of VOCs and their synergistic interactions with other environmental factors have yet to be elucidated; and (3) a systematic analysis of a VOC-driven micro-ecological reconstruction of the root microbiome is lacking, which restricts their field application potential. Furthermore, variations in the types and quantities of VOCs produced by different PGPR strains, as well as their growth-promoting effects on cherry seedlings, require further validation and optimization.

This study investigates the regulatory mechanisms of VOCs produced by three high-efficiency PGPR strains (*Pantoea ananatis* D1-28, *Burkholderia* sp. D4-24, and *Burkholderia territorii* D4-36) on seedling growth using the cherry rootstock ‘Gisela 6′ as the model system. The core research objectives were to (1) elucidate the impact of VOCs on cherry seedling growth and root auxin biosynthesis gene expression; (2) decipher the patterns of VOC-mediated rhizosphere microbial community succession; and (3) validate the scientific hypothesis of VOCs′ synergistic regulation of plant growth through a “hormone-microbiome” dual pathway. The theoretical significance lies in clarifying the cross-scale regulatory mechanisms of PGPR-VOCs and their translatability from laboratory observations to complex field environments. These insights will establish new paradigms for rhizosphere micro-ecological management and advance the transformation of PGPR-VOC research from mechanistic dissection to application design.

## 2. Materials and Methods

### 2.1. Test Strains and Plants

The bacterial strains used in this experiment, *Pantoea ananatis* D1-28, *Burkholderia* sp. D4-24, and *Burkholderia territorii* D4-36, were deposited at the China General Microbiological Culture Collection Center (CGMCC) under accession numbers CGMCC 24784, 30613, and 30614, respectively. For inoculum preparation, each test strain (D1-28, D4-24, and D4-36) was initially cultured on a beef extract peptone agar medium (0.3% beef extract, 0.5% sodium chloride, 1% peptone, 2% agar, and pH 7.0–7.4) at 37 °C for 24 h. Single colonies were then picked and transferred to 50 mL of a beef extract peptone liquid medium in a flask, followed by incubation at 37 °C with shaking at 180 rpm for 12 h to obtain the seed culture.

We selected one of the most popular sweet cherry rootstocks in China, Gisela 6, as the plant material. Experimental seedlings were propagated and rooted through plant tissue culture techniques. The planting soil was taken from the test field of the Haicheng Scientific Research Base of Shenyang Agricultural University. Subsequently, the soil was mixed with sand at a 2:1 ratio, with available nitrogen (AN), phosphorus (AP), potassium (AK), and organic carbon (SOC) contents of 75.78 mg/kg, 112.35 mg/kg, 175.46 mg/kg, and 11.79 g/kg, respectively. Uniform-growth Gisela 6 tissue culture seedlings were selected and transplanted into plastic pots containing non-sterilized mixed soil. The experimental seedlings grew naturally in the fruit tree scientific research base and were watered every two days. Seedlings were treated when they reached approximately 10 cm in height, a stage where cherry stems achieve semi-lignification, exhibiting enhanced mechanical stress resistance and significantly higher transplantation survival rates (typically >85%, based on our preliminary trials).

### 2.2. Co-Cultivation of Strains with Plants

**Figure 1:**
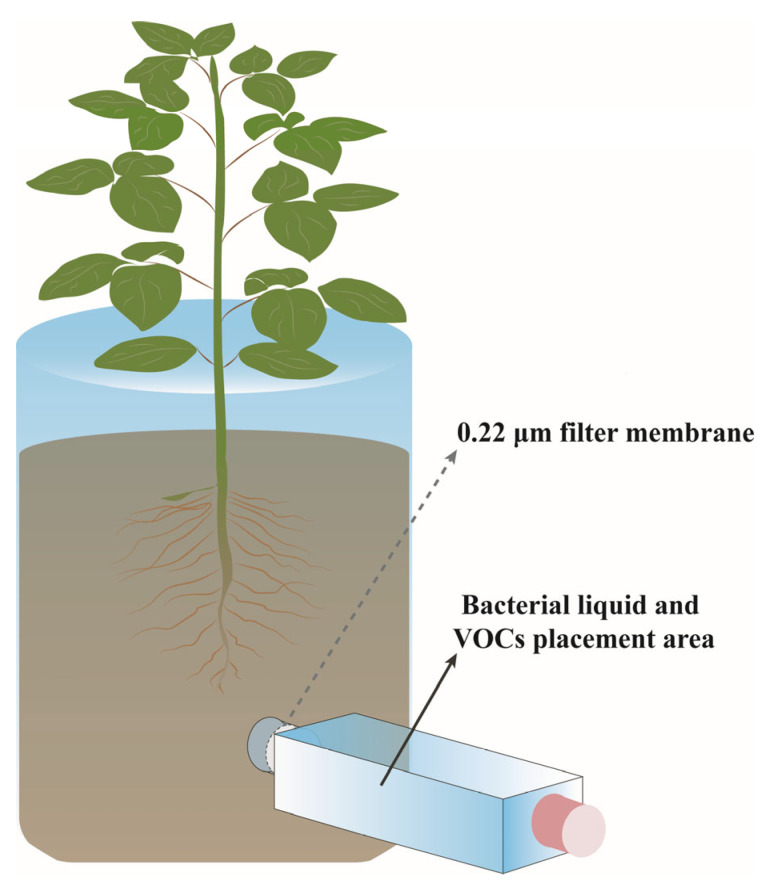
Schematic diagram of root olfactometer.

As shown in Figure 1, co-culture experiments were performed in a root olfactory apparatus, which was designed according to the apparatus described by Kallenbach et al. and Ryu et al. [27,28]. The root olfactometer primarily consists of two main components, the main body and the arm terminal. The main body serves as the growth region for plants, while the arm terminal is designated for the application of bacterial fermentation solutions. The connection between the main body and the arm terminal is filled with sponge material to prevent soil particle movement that could compromise the isolation between these two sections. Additionally, a 0.22 μm pore size filter membrane is placed at the tip of the arm terminal to prevent microbial migration between the main body and the arm terminal. Finally, the end of the arm is covered with a lid to prevent gas from escaping.

First, cherry seedlings with a height of approximately 10 cm were selected and transplanted into the root olfactory devices equipped with soil, with one seedling per device and three biological replicates per set-up. Subsequently, 1 mL of the D1-28, D4-24, and D4-36 bacterial cultures, each cultured for 12 h, was taken and placed into a 24-well cell culture plate. The OD_600 nm_ value was adjusted to 0.4 using a CYTATION 3 microplate reader (BioTek, Winooski, VT, USA), followed by dilution with distilled water to create eight treatment groups with bacterial concentrations of 10^8^, 10^7^, 10^6^, 10^5^, 10^4^, 10^3^, and 10^2^ cfu/mL, respectively. The control group received distilled water without a bacterial inoculum. The diluted bacterial solutions were then injected into the tip of the root olfactory device′s arm end. Finally, the inoculated root olfactory devices were placed in a light-controlled incubator for 60 days, with a light/dark cycle of 2/1, a temperature setting of 25/16 °C, and humidity maintained at 50–60% RH.

### 2.3. Determination of Plant Growth Indicators

Seedling height was measured using a tape measure and a vernier caliper. Parameters such as root length, root surface area, and root volume of the cherry seedlings were determined using a STD4800 Scanner root-scanning system. Shoot and root biomass data of the cherry seedlings were ascertained using an SQP electronic balance.

### 2.4. Determination of Gene Expression Levels Related to Auxin Biosynthesis in Cherry Roots

Following treatment with different bacterial strains, cherry root samples from various treatment groups were collected for RNA extraction. Total RNA was isolated using the column-based Universal Total RNA Purification Kit (Sangon Biotech Co., Ltd., Shanghai, China) following the manufacturer′s protocol. Subsequently, a quantitative real-time PCR (qPCR) analysis of auxin-biosynthesis-related genes in cherry was performed using a QuantStudio 6 Flex system (Thermo Fisher Scientific, Waltham, MA, USA) employing the relative quantification method (2^−ΔΔCT^). The experiments utilized the SYBR Green Premix Pro Taq HS qPCR Kit (AG11718, Accurate Biotechnology Co., Ltd., Changsha, China) according to the manufacturer′s instructions. *PavRSP3* was selected as the internal reference gene. The primer sequences and reaction mixtures are detailed in Table S1.

### 2.5. Microbial Diversity and Community Composition

Following 60 days of co-culture with different bacterial strains, root-associated soil samples from a 10–15 cm deep layer in the root olfactory device were collected. Residual cherry roots were removed from the soil samples, which were then stored at −80 °C before being sent to Shanghai Personal Biotechnology Co., Ltd, Shanghai, China for high-throughput sequencing using the Illumina platform. Total DNA was extracted from cherry-root-associated soils treated with the D1-28, D4-24, and D4-36 strains. The following specific primers were used for amplification and library construction: 338F (5′-ACTCCTACGGGAGGCAGCA-3′) and 806R (5′-GGACTACHVGGGTWTCTAAT-3′) for the *16S rRNA* gene analysis. The bioinformatics pipeline was adapted from QIIME2, version 2019.4, following official tutorials. In QIIME2, we applied the following quality control criteria. Sequences were truncated at the first occurrence of 10 consecutive low-quality (Phred < Q30) bases in either direction (--p-trunc-len-f/r parameters). Reads with a final length < 150 bp or containing ambiguous bases were discarded. We dereplicated sequences using the qiime feature-table summarize command to collapse exact duplicates. Chimeric sequences were identified and removed using the q2-dada2 plugin with default hyperparameter settings (--p-chimera-method ‘consensus’)”. Taxonomic classification was performed against the Greengenes 13_8 99% OTU database using the q2-feature-classifier plugin with a naively Bayesian classifier trained on sequences from the V3-V4 region (--p-reads-per-batch 10,000 --p-confidence 0.7). The DADA2 plugin was employed for quality filtering, denoising, merging, and chimera removal. Amplicon sequence variants (ASVs) and abundance tables were generated after sequence merging. Raw sequencing data were deposited in the NCBI Sequence Read Archive (SRA) under accession number PRJNA1262684.

### 2.6. Statistical Analysis

A statistical analysis of the experimental data was performed using IBM SPSS 25.0 software (SPSS Inc., Chicago, IL, USA). All experiments were conducted in triplicate, and the data were presented as the mean ± standard error (SE). A one-way analysis of variance (ANOVA), followed by Tukey’s test for multiple comparisons, was used to analyze the differences among treatments, with statistical significance set at *p* < 0.05.

## 3. Results

### 3.1. Effects of VOCs Produced by Different Bacterial Strains on the Growth of Cherry Seedlings

**Figure 2:**
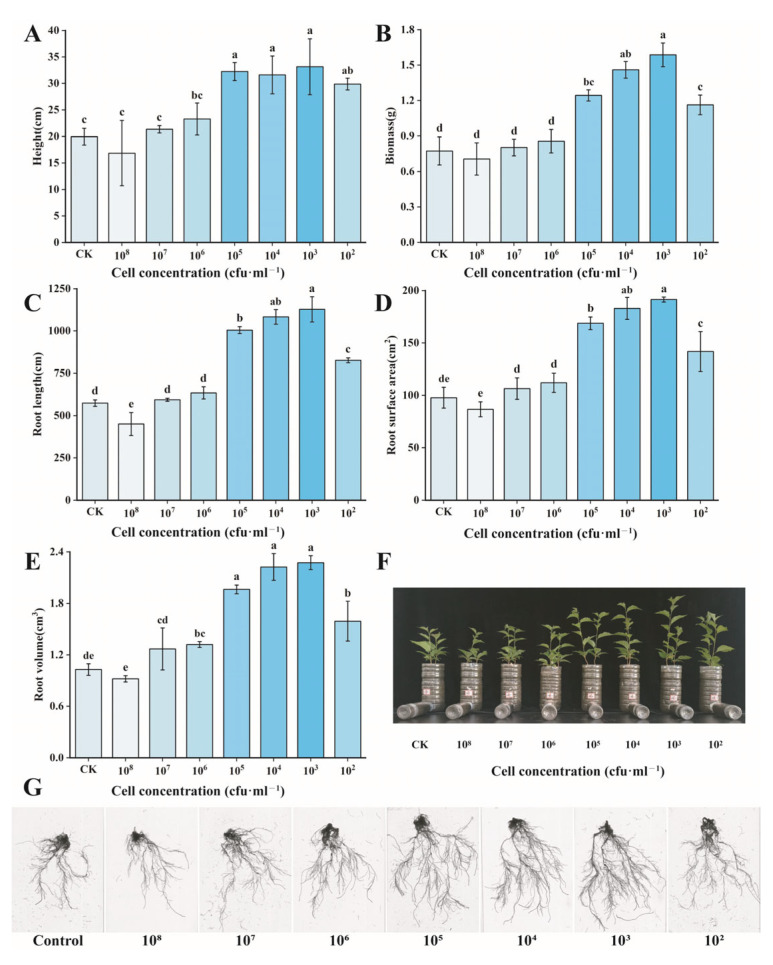
Effects of different concentrations of *P. ananatis* D1-28 bacterial suspension treatment on cherry growth. (**A**) Height; (**B**) biomass; (**C**) root length; (**D**) root surface area; (**E**) root volume; (**F**) phenotypic changes in cherry seedlings; (**G**) root-scanning image of cherry seedlings. All data are from three independent biological replicate means ± SE (*n* = 3). Different letters indicate the degree of significant difference between treatments. Values with the same letter above the bar graph indicate no significant difference according to the Tukey test.

The experimental results showed that the volatile organic compounds (VOCs) produced by the three bacterial strains D1-28, D4-24, and D4-36 had significant growth-promoting effects on cherry seedlings. Compared with the control, the optimal growth-promoting effect on cherry seedlings was observed when the bacterial count of D1-28 was at 10^3^ cfu/mL (Figure 2). The average plant height of cherry seedlings increased by 66% (from 19.95 cm to 33.13 cm), total biomass increased by 488% (from 0.27 g to 1.59 g), and root length, root surface area, and root volume significantly increased by 97% (573.75 → 1127.73 mm), 96% (97.75 → 191.39 cm^2^), and 121% (1.03 → 2.27 cm^3^), respectively.

**Figure 3:**
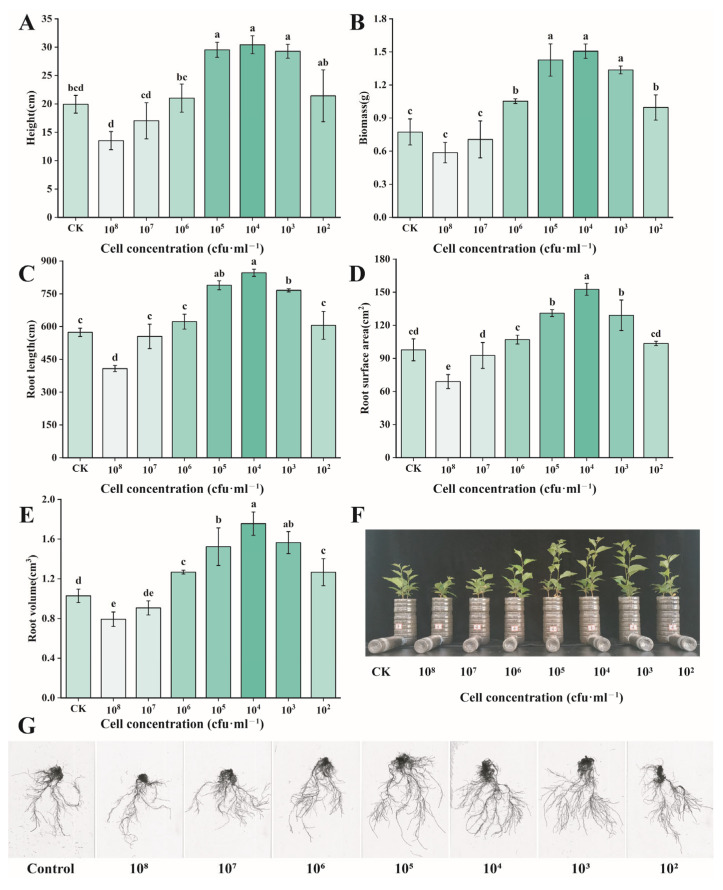
Effects of different concentrations of *Burkholderia* sp. D4-24 bacterial suspension treatment on cherry growth. (**A**) Height; (**B**) biomass; (**C**) root length; (**D**) root surface area; (**E**) root volume; (**F**) phenotypic changes in cherry seedlings; (**G**) root-scanning image of cherry seedlings. All data are from three independent biological replicate means ± SE (*n* = 3). Different letters indicate the degree of significant difference between treatments. Values with the same letter above the bar graph indicate no significant difference according to the Tukey test.

When cultured with cherry seedlings for 60 days using different concentrations of *Burkholderia* sp. D4-24, the results showed that, at a bacterial count of 10^3^ cfu/mL, the average plant height of cherry seedlings increased by 53% (19.95 → 30.43 cm), total biomass increased by 395% (0.27 → 1.34 g), and root length, root surface area, and root volume increased by 47% (573.75 → 846.19 mm), 56% (97.75 → 152.59 cm^2^), and 71% (1.03 → 1.76 cm^3^), respectively (Figure 3).

**Figure 4:**
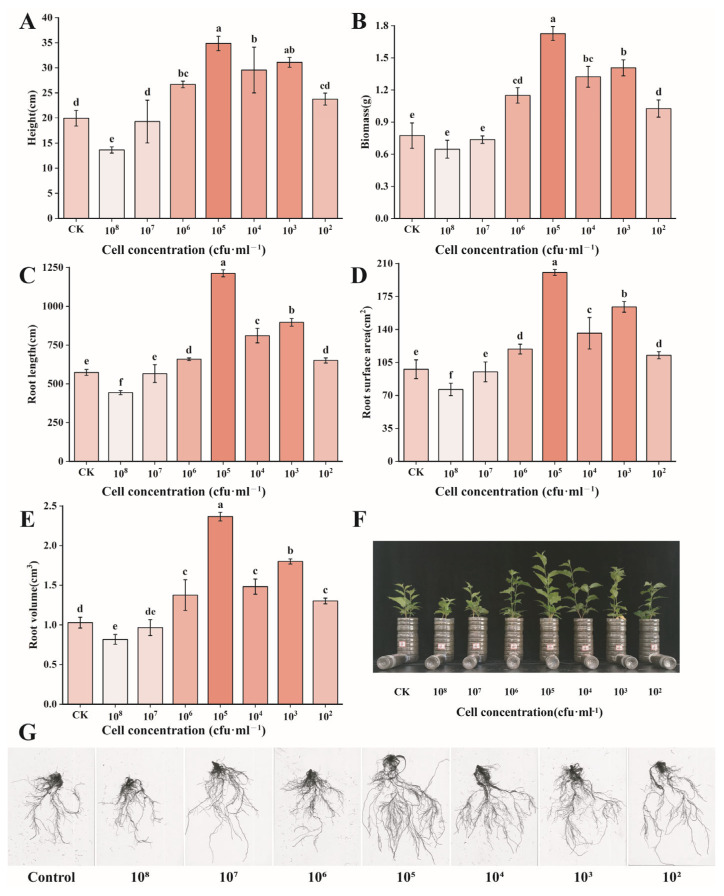
Effects of different concentrations of *B. territorii* D4-36 bacterial suspension treatment on cherry growth. (**A**) Height; (**B**) biomass; (**C**) root length; (**D**) root surface area; (**E**) root volume; (**F**) phenotypic changes in cherry seedlings; (**G**) root-scanning image of cherry seedlings. All data are from three independent biological replicate means ± SE (*n* = 3). Different letters indicate the degree of significant difference between treatments. Values with the same letter above the bar graph indicate no significant difference according to the Tukey test.

After co-culturing cherry seedlings with different concentrations of *B. territorii* D4-36 for 60 days, the root architecture of the seedlings was significantly altered, with notable increases in the root length and root surface area (Figure 4). At a bacterial count of 10^5^ cfu/mL, the average plant height of cherry seedlings increased by 75% (19.95 → 34.87 cm), total biomass increased by 540% (0.27 → 1.73 g), and root length, root surface area, and root volume increased by 111% (573.75 → 1212.59 mm), 105% (97.75 → 200.55 cm^2^), and 130% (1.03 → 2.37 cm^3^), respectively.

### 3.2. Effects of VOCs Produced by Different Bacterial Strains on the Expression of Auxin-Biosynthesis-Related Genes in Cherry Roots

**Figure 5:**
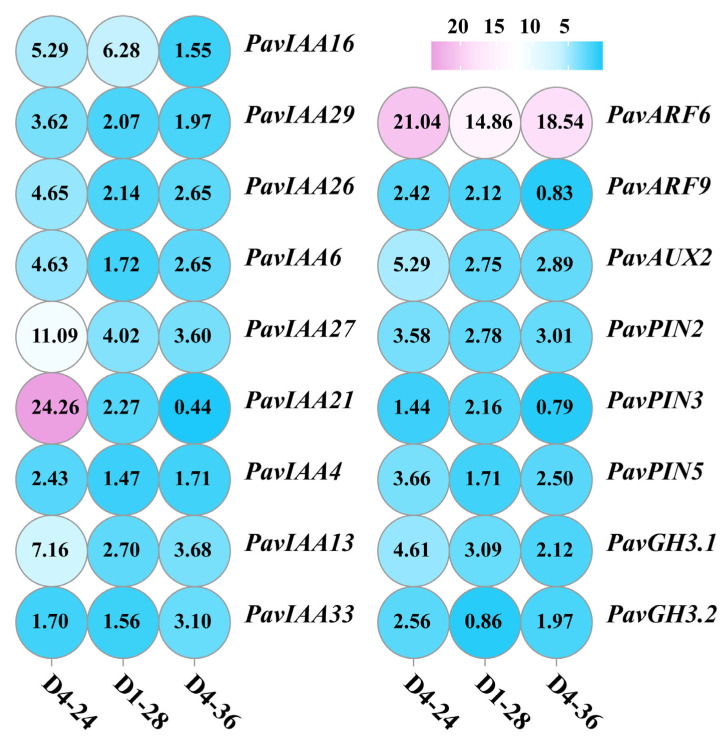
Effect of bacterial VOC treatment on the expression levels of auxin -synthesis-related genes in cherry roots.

Through co-culturing three different plant growth-promoting bacterial strains, we co-cultured three PGPR strains with cherry seedlings under optimal growth-promoting concentration conditions, and we ultimately detected changes in the expression levels of genes related to auxin biosynthesis, metabolism, transport, and regulation in cherry roots. The results showed that after co-culturing with cherry seedlings, the three growth-promoting bacterial strains upregulated the expression of auxin-responsive protein genes *PavIAA4*, *PavIAA6*, *PavIAA13*, *PavIAA16*, *PavIAA21*, *PavIAA26*, *PavIAA27*, *PavIAA29*, and *PavIAA33*; auxin transcription factors *PavARF6* and *PavARF9*; the auxin efflux protein *PavAUX2*; auxin transporter proteins *PavPIN1*, *PavPIN2*, and *PavPIN5*; and auxin metabolism genes *PavGH3.1* and *PavGH3.2* (Figure 5).

### 3.3. Effects of VOCs Produced by Different Bacterial Strains on Bacterial Diversity in Cherry Rhizosphere Soil

**Figure 6:**
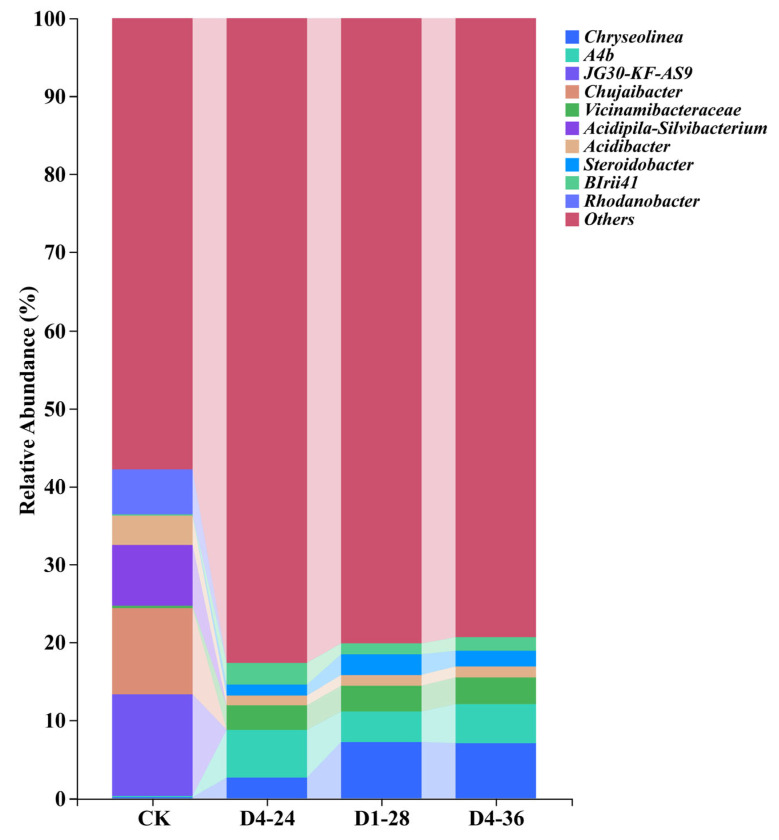
Effect of bacterial VOC treatment on the microbial community in cherry roots.

The results indicate that, in terms of bacterial diversity, the three bacteria with the highest relative abundance in the control group were *JG30-KF-AS9* (8.03%), *Chujaibacter* (7.95%), and *Acidipila-Silvibacterium* (6.49%) (Figure 6). In contrast, the microbial community structure in the treatment groups underwent significant changes. After treatment with three distinct PGPR strains (*P. ananatis* D1-28, *Burkholderia* sp. D4-24, and *B. territorii* D4-36), the top three bacteria with the highest relative abundance in the cherry-root-associated soil shifted to *A4b*, *Chryseolinea*, and *Vicinamibacteraceae*. Specifically, in the D1-28 treatment group, the relative abundances of these dominant bacteria were 3.90%, 7.26%, and 3.29%, respectively; in the D4-24 treatment group, they were 6.06%, 2.71%, and 3.13%, respectively; and in the D4-36 treatment group, they were 4.94%, 7.13%, and 3.48%, respectively. The data show that the three bacteria with a high relative abundance in the control group—*JG30-KF-AS9*, *Chujaibacter*, and *Acidipila-Silvibacterium*—almost disappeared in the treatment groups, while the relative abundance of *A4b*, *Chryseolinea*, *Vicinamibacteraceae*, and *Steroidobacter* significantly increased. Additionally, an α-diversity analysis revealed that the Chao1 and Shannon indices of the D1-28, D4-24, and D4-36 treatment groups were significantly higher than those of the control group (Figure S1). This indicates that the bacterial VOC treatments substantially enhanced the richness of bacterial communities, leading to a significant increase in bacterial diversity in the root-associated soil of cherry seedlings.

## 4. Discussion

PGPR have garnered increasing attention in sustainable agriculture due to their efficient, persistent, and eco-friendly growth-promoting properties [11,13,29]. Their VOCs, characterized by a broad action range, low effective concentrations, and minimal environmental impact, enable the long-distance regulation of plant growth while stably enhancing plant biomass and resistance to biotic/abiotic stresses, as extensively reported [30]. Previous research on bacterial VOCs has predominantly focused on model plants such as *Arabidopsis thaliana* and *Nicotiana benthamiana* [25,31]. For instance, Ryu et al. (2003) first demonstrated that VOCs from *Bacillus subtilis* GB03 and *Bacillus amyloliquefaciens* IN937a significantly promote shoot development in *Arabidopsis* [28]. Similarly, Zou et al. (2010) reported that volatiles emitted by *Bacillus megaterium* XTBG-34 increased the fresh weight of *Arabidopsis* 1.7-fold after 7 days of exposure [32]. In this study, we investigated the growth-promoting effects of VOCs from three bacterial strains—*P. ananatis* D1-28, *Burkholderia* sp. D4-24, and *B. territorii* D4-36—on cherry seedlings. Our findings demonstrate that VOCs from all three strains significantly enhanced cherry seedling growth, expanding the application scope of PGPR-VOCs to woody fruit crops and providing novel insights for orchard management.

As an important class of growth-promoting substances, VOCs pose challenges when determining optimal concentrations due to uncertainties in their dosage and compositional diversity [33]. Previous studies have demonstrated that VOCs exert variable effects on plant growth, with nonlinear relationships observed between concentration and growth promotion [34]. Although high VOC concentrations may inhibit plant development, appropriate levels prove beneficial [35]. Therefore, the precise control of VOC concentrations is critical for maximizing their biostimulative potential. Our results further reveal that the three PGPR strains—*P. ananatis* D1-28, *Burkholderia* sp. D4-24, and *B. territorii* D4-36—exhibited peak growth-promoting effects on cherry plants when cultured at bacterial concentrations of 10^3^, 10^3^, and 10^5^ cfu/mL, respectively.

The primary mechanism by which bacterial VOCs promote plant growth is likely to be closely associated with auxin synthesis [36,37]. Auxin, a class of widely distributed plant hormones, plays a crucial role in regulating various developmental stages of plants [38]. By binding to receptor components on cell membranes or intracellular elements and interacting with other signal transduction pathways, auxin directly modulates cell division, differentiation, and elongation processes, thereby orchestrating plant development [39,40]. Numerous gene families participate in auxin signaling transduction, including Auxin Response Factors (ARFs), Auxin/Indole-3-Acetic Acid (AUX/IAA), Small Auxin-UpRNAs (SAURs), and Gretchen Hagen3 (GH3) families [40,41]. In plants, auxin levels are dynamic rather than static. Fluctuations in auxin concentrations often trigger transient expression changes in these gene families, enabling plants to adjust their growth and development while responding to environmental variations [42]. In this study, we observed that under optimal growth-promoting concentrations, the co-cultivation of three PGPR strains with cherry seedlings induced the significant upregulation of genes associated with IAA-responsive proteins, transcription factors, and efflux carriers [43,44]. This indicates that VOCs emitted by these strains enhance auxin sensitivity in cherry seedlings, thereby promoting elevated IAA synthesis in roots to further stimulate growth. Furthermore, bacterial VOCs directly modulate cherry root architecture by altering the relocation of PIN-FORMED (PIN) efflux carriers, the plant-specific proteins governing auxin transport. Notably, this PIN-mediated root remodeling may occur independently of auxin biosynthesis pathways [45,46].

In addition to directly promoting root growth by upregulating auxin biosynthesis genes in cherry plants, VOCs produced by PGPR also indirectly enhance plant growth by restructuring the rhizosphere microbial community. This remodeling drives the functional reprogramming of the microbiome, establishing a functionally synergistic micro-ecosystem that supports plant development [47]. Our study revealed that a PGPR-VOC treatment significantly increased the abundance of specific microbial taxa in cherry root soil, including *A4b*, *Chryseolinea*, *Vicinamibacteraceae*, *Steroidobacter*, *s0134_terrestral_group*, and *SM1A02*. Among these, the oligotrophic bacterium *A4b* may suppress pathogen proliferation (e.g., Fusarium) under low-nutrient conditions through competitive inhibition [48]. *Chryseolinea*, known for degrading complex organics like lignin, accelerates soil organic matter mineralization to release soluble phosphorus and nitrogen [49]. Increased *Vicinamibacteraceae* abundance enhances plant tolerance to iron deficiency and heavy metal stress [50]. *Steroidobacter* inhibits phytopathogenic fungi by degrading their cell walls while releasing carbon sources for symbiotic microbes [50,51]. Taxa such as *s0134_terrestral_group* and *SM1A02* participate in denitrification to maintain root sulfur homeostasis [51,52]. By modulating the activity of functional taxa like *A4b* and *Chryseolinea*, bacterial VOCs create a cascading effect—”chemical signaling → microbial function → plant growth”—that synergistically promotes cherry development through both direct and indirect pathways. Under PGPR-VOC treatment, although these functional groups were significantly enriched, the translatability of these effects to field conditions remains uncertain. Field ecosystems are influenced by dynamic abiotic fluctuations (e.g., temperature, humidity, and soil pH) and biotic interactions (e.g., competition with indigenous microbiota and plant–soil feedback), all of which may alter VOC efficacy or microbial stability. For instance, volatile organic compounds with lower persistence (e.g., alcohols and ketones) may rapidly volatilize in porous soil, limiting their spatiotemporal impact. Furthermore, the resilience of VOC-altered communities—their ability to maintain functionality under environmental stresses (e.g., drought and salinity)—requires investigation. Future research should prioritize field-scale mesocosm trials or long-term orchard experiments using VOC-emitting PGPR inoculants to assess ecological robustness and sustainability. Although PGPR-VOCs promoted growth and beneficial microbial taxa in vitro, their long-term impacts on microbial alpha/beta diversity and plant physiology require scrutiny. High concentrations of certain VOCs could inhibit soil enzyme activities or disrupt mycorrhizal symbioses, offsetting the perceived benefits.

In summary, our results demonstrate that bacterial VOCs form a triadic interaction network—“plant-VOCs-microbes”—by influencing plant hormone synthesis and reshaping the structure and function of root-associated microbial communities, providing theoretical support for eco-friendly agriculture. However, this study only utilized three PGPR strains; different PGPR lineages may produce distinct volatile organic compounds, which could trigger varying microbial responses. Moreover, the lack of identification of specific bioactive compounds leaves their mechanisms speculative. For instance, unidentified VOCs might act as quorum-sensing inhibitors or inducers, altering microbial behavior independently of plant hormones.

Future research should integrate pangenomic or metabolomic comparisons across diverse PGPR strains to distinguish conserved versus cherry-specific effects on the root microbiome while combining comprehensive VOC profiling (e.g., GC-MS and SPME-GC/MS) with dose–response assays to link chemical identities with ecological functions. Finally, comparative studies using multi-species root-box systems (e.g., peach and apple) or synthetic media mimicking root exudates could clarify whether PGPR-VOC priming represents a universal strategy or is tailored to the chemical ecology of the cherry. By addressing these gaps, we can refine PGPR-VOC technology to harness the plasticity of root microbiomes for sustainable cherry cultivation in diverse agroecosystems.

## 5. Conclusions

Under controlled laboratory conditions, the volatile organic compounds (VOCs) produced by three PGPR strains, D1-28, D4-24, and D4-36, significantly increased the biomass of Gisela 6 cherry rootstock. The optimal growth-promoting effects of the VOCs on the cherry were observed at bacterial concentrations of 10^3^, 10^3^, and 10^5^ cfu·mL^−1^ for strains D1-28, D4-24, and D4-36, respectively. The VOCs produced by these PGPR strains may regulate auxin synthesis in cherry roots, promoting increased IAA production and significantly altering the composition of the microbial community in the root environment to further promote cherry seedling growth. This study provides valuable in vitro evidence and expands the PGPR database by identifying strains with VOC-mediated growth-promotion potential. It offers new theoretical insights into the mechanisms, including hormonal modulation and microbiome remodeling, by which PGPR VOCs may enhance cherry root growth. Future research should prioritize (i) identifying the specific bioactive volatile organic compounds; (ii) validating these effects across a broader range of PGPR strains and cherry varieties; and (iii) conducting field trials to assess the robustness, sustainability, and practical applicability of using VOC-emitting PGPR in cherry cultivation.

## Data Availability

The original contributions presented in the study are included in the article/Supplementary Materials, further inquiries can be directed to the corresponding authors.

## Supplementary Materials

The following supporting information can be downloaded at: https://www.mdpi.com/article/10.3390/foods14132369/s1, Figure S1: Effect of bacterial VOCs treatment on the bacterial alpha diversity index (Genus level) in cherry roots; Table S1: Primers for quantitative real-time PCR in this study.

## References

[1] Prinsi B., Negri A.S., Espen L., Piagnani M.C. (2016). Proteomic comparison of fruit ripening between ‘Hedelfinger’ sweet cherry (*Prunus avium* L.) and its somaclonal variant ‘HS’. J. Agric. Food Chem..

[2] Zhou W., Qin X., Lyu D., Qin S. (2021). Effect of glucose on the soil bacterial diversity and function in the rhizosphere of *Cerasus sachalinensis*. Hortic. Plant J..

[3] Yuri J.A., Simeone D., Fuentes M., Sepúlveda Á., Palma M., Moya M., Sánchez-Contreras J. (2024). Reduced Root Volume at Establishment, Canopy Growth and Fruit Production in ‘Lapins’/‘Colt’ and ‘Regina’/‘Gisela 12’ Sweet Cherry Trees. Horticulturae.

[4] Wedegaertner K., Black B., Safre A., Lilligren C., Cardon G., Torres-Rua A. (2023). Assessing the relationship between soil variability, canopy density, and yield in Utah tart cherry orchards. Acta Hortic..

[5] Guo Y., Wang J. (2021). Spatiotemporal changes of chemical fertilizer application and its environmental risks in China from 2000 to 2019. Int. J. Environ. Res. Public Health.

[6] Pahalvi H.N., Rafiya L., Rashid S., Nisar B., Kamili A.N., Dar G.H., Bhat R.A., Mehmood M.A., Hakeem K.R. (2021). Chemical Fertilizers and Their Impact on Soil Health. Microbiota and Biofertilizers, Vol 2: Ecofriendly Tools for Reclamation of Degraded Soil Environs.

[7] Wang P., Zhang W., Zhu Y., Liu Y., Li Y., Cao S., Hao Q., Liu S., Kong X., Han Z. (2024). Evolution of groundwater hydrochemical characteristics and formation mechanism during groundwater recharge: A case study in the Hutuo River alluvial–pluvial fan, North China Plain. Sci. Total Environ..

[8] Albini D., Lester L., Sanders P., Hughes J., Jackson M.C. (2023). The combined effects of treated sewage discharge and land use on rivers. Glob. Change Biol..

[9] Sun B., Zhang L., Yang L., Zhang F., Norse D., Zhu Z. (2012). Agricultural non-point source pollution in China: Causes and mitigation measures. AMBIO.

[10] Edenhofer O. (2015). Climate Change 2014: Mitigation of Climate Change.

[11] Deng C., Zhang N., Liang X., Huang T., Li B. (2022). *Bacillus aryabhattai* LAD impacts rhizosphere bacterial community structure and promotes maize plant growth. J. Sci. Food. Agric..

[12] Deng C., Liang X., Zhang N., Li B., Wang X., Zeng N. (2022). Molecular mechanisms of plant growth promotion for methylotrophic *Bacillus aryabhattai* LAD. Front. Microbiol..

[13] Deng C., Zeng N., Li C., Pang J., Zhang N., Li B. (2024). Mechanisms of ROS-mediated interactions between *Bacillus aryabhattai* LAD and maize roots to promote plant growth. BMC Microbiol..

[14] Prakash R., Subramani R., Krodi, Anusha, Berde C.V., Chandrasekhar T., Prathyusha A.M.V.N., Kariali E., Bramhachari P.V., Veera Bramhachari P. (2022). Rhizobacteriome: Plant Growth-Promoting Traits and Its Functional Mechanism in Plant Growth, Development, and Defenses. Understanding the Microbiome Interactions in Agriculture and the Environment.

[15] Kapadia C., Patel N., Rana A., Vaidya H., Alfarraj S., Ansari M.J., Gafur A., Poczai P., Sayyed R.Z. (2022). Evaluation of Plant Growth-Promoting and Salinity Ameliorating Potential of Halophilic Bacteria Isolated From Saline Soil. Front. Plant Sci..

[16] He Y., Guo W., Peng J., Guo J., Ma J., Wang X., Zhang C., Jia N., Wang E., Hu D. (2022). Volatile Organic Compounds of *Streptomyces* sp. TOR3209 Stimulated Tobacco Growth by Up-Regulating the Expression of Genes Related to Plant Growth and Development. Front. Microbiol..

[17] Jiang L., Lee M.H., Kim C.Y., Kim S.W., Kim P.I., Min S.R., Lee J. (2021). Plant Growth Promotion by Two Volatile Organic Compounds Emitted From the Fungus *Cladosporium halotolerans* NGPF1. Front. Plant Sci..

[18] Tilocca B., Cao A., Migheli Q. (2020). Scent of a Killer: Microbial Volatilome and Its Role in the Biological Control of Plant Pathogens. Front. Microbiol..

[19] Li P., Kong W., Wu X., Zhang Y. (2021). Volatile Organic Compounds of the Plant Growth-Promoting Rhizobacteria JZ-GX1 Enhanced the Tolerance of *Robinia pseudoacacia* to Salt Stress. Front. Plant Sci..

[20] Zhao D., Jiao J., Du B., Liu K., Wang C., Ding Y. (2022). Volatile organic compounds from *Lysinibacillus macroides* regulating the seedling growth of Arabidopsis thaliana. Physiol. Mol. Biol. Plants.

[21] Zheng D., Ren F., Yin R., Li L., Cui H., Shen H., Chen X., Gao N. (2025). Transcriptome Analysis Reveals Impact of S-Methyl Thioacetate on Tomato Seedling Growth. J. Plant Growth Regul..

[22] Kumar D., Ali M., Sharma N., Sharma R., Manhas R.K., Ohri P. (2024). Unboxing PGPR-mediated management of abiotic stress and environmental cleanup: What lies inside?. Environ. Sci. Pollut. Res..

[23] Cai Y., Tao H., Gaballa A., Pi H., Helmann J.D. (2025). The extracytoplasmic sigma factor σ^X^ supports biofilm formation and increases biocontrol efficacy in *Bacillus velezensis* 118. Sci. Rep..

[24] Velázquez-Becerra C., Macías-Rodríguez L.I., López-Bucio J., Altamirano-Hernández J., Flores-Cortez I., Valencia-Cantero E. (2011). A volatile organic compound analysis from *Arthrobacter agilis* identifies dimethylhexadecylamine, an amino-containing lipid modulating bacterial growth and *Medicago sativa* morphogenesis in vitro. Plant Soil.

[25] Hung R., Lee S., Bennett J.W. (2013). *Arabidopsis thaliana* as a model system for testing the effect of *Trichoderma* volatile organic compounds. Fungal Ecol..

[26] Del Carmen Orozco-Mosqueda M., Velázquez-Becerra C., Macías-Rodríguez L.I., Santoyo G., Flores-Cortez I., Alfaro-Cuevas R., Valencia-Cantero E. (2013). *Arthrobacter agilis* UMCV2 induces iron acquisition in *Medicago truncatula* (strategy I plant) in vitro via dimethylhexadecylamine emission. Plant Soil.

[27] Kallenbach M., Oh Y., Eilers E.J., Veit D., Baldwin I.T., Schuman M.C. (2014). A robust, simple, high-throughput technique for time-resolved plant volatile analysis in field experiments. Plant J..

[28] Ryu C., Farag M.A., Hu C., Reddy M.S., Wei H., Paré P.W., Kloepper J.W. (2003). Bacterial volatiles promote growth in *Arabidopsis*. Proc. Natl. Acad. Sci. USA.

[29] Zipfel C. (2014). Plant pattern-recognition receptors. Trends Immunol..

[30] Liu S., Xie J., Luan W., Liu C., Chen X., Chen D. (2024). *Papiliotrema flavescens*, a plant growth-promoting fungus, alters root system architecture and induces systemic resistance through its volatile organic compounds in *Arabidopsis*. Plant Physiol. Biochem..

[31] Meldau D.G., Meldau S., Hoang L.H., Underberg S., Wünsche H., Baldwin I.T. (2013). Dimethyl Disulfide Produced by the Naturally Associated Bacterium *Bacillus* sp. B55 Promotes *Nicotiana attenuata* Growth by Enhancing Sulfur Nutrition. Plant Cell.

[32] Zou C., Li Z., Yu D. (2010). *Bacillus megaterium* strain XTBG34 promotes plant growth by producing 2-pentylfuran. J. Microbiol..

[33] Fincheira P., Quiroz A. (2018). Microbial volatiles as plant growth inducers. Microbiol. Res..

[34] Raza W., Jiang G., Eisenhauer N., Huang Y., Wei Z., Shen Q., Kowalchuk G.A., Jousset A. (2024). Microbe-induced phenotypic variation leads to overyielding in clonal plant populations. Nat. Ecol. Evol..

[35] Xia H., Liu H., Gong P., Li P., Xu Q., Zhang Q., Sun M., Meng Q., Ye F., Yin W. (2025). Study of the mechanism by which *Bacillus subtilis* improves the soil bacterial community environment in severely saline-alkali cotton fields. Sci. Total Environ..

[36] Tzipilevich E., Russ D., Dangl J.L., Benfey P.N. (2021). Plant immune system activation is necessary for efficient root colonization by auxin-secreting beneficial bacteria. Cell Host Microbe.

[37] Jiang C., Xie Y., Zhu K., Wang N., Li Z., Yu G., Guo J. (2019). Volatile organic compounds emitted by *Bacillus* sp. JC03 promote plant growth through the action of auxin and strigolactone. Plant Growth Regul..

[38] Casanova-Sáez R., Voß U. (2019). Auxin metabolism controls developmental decisions in land plants. Trends Plant Sci..

[39] Peer W.A. (2013). From perception to attenuation: Auxin signalling and responses. Curr. Opin. Plant Biol..

[40] Hagen G. (2015). Auxin signal transduction. Essays Biochem..

[41] Qiu T., Qi M., Ding X., Zheng Y., Zhou T., Chen Y., Han N., Zhu M., Bian H., Wang J. (2020). The *SAUR41* subfamily of *SMALL AUXIN UP RNA* genes is abscisic acid inducible to modulate cell expansion and salt tolerance in Arabidopsis thaliana seedlings. Ann. Bot..

[42] Lavy M., Estelle M. (2016). Mechanisms of auxin signaling. Development.

[43] Guilfoyle T.J., Hagen G. (2007). Auxin response factors. Curr. Opin. Plant Biol..

[44] Chandler J.W. (2016). Auxin response factors. Plant Cell Environ..

[45] Zwiewka M., Bilanovičová V., Seifu Y.W., Nodzyński T. (2019). The Nuts and Bolts of PIN Auxin Efflux Carriers. Front. Plant Sci..

[46] Wojtaczka P., Ciarkowska A., Starzynska E., Ostrowski M. (2022). The GH3 amidosynthetases family and their role in metabolic crosstalk modulation of plant signaling compounds. Phytochemistry.

[47] Wang J., Mei X., Wei Z., Raza W., Shen Q. (2021). Effect of bacterial intra-species community interactions on the production and activity of volatile organic compounds. Soil Ecol. Lett..

[48] He Q., Zhang Q., Li M., He J., Lin B., Wu N., Chen J., Liu X., Dong X. (2025). Harnessing diurnal dynamics: Understanding the influence of light–dark cycle on algal-bacterial symbiotic system under aniline stress. Bioresour. Technol..

[49] Zhou Z., Wang X., Chen C., Cui Z., Li A., He W., Guo Y., Zeng Y. (2025). Agroforestry system: *Polygonatum odoratum* and *Vernicia fordii* intercropping effects on crop quality, soil nutrients and microbial community structure. Agrofor. Syst..

[50] Zhou X., Zhang J., Shi J., Khashi U Rahman M., Liu H., Wei Z., Wu F., Dini-Andreote F. (2024). Volatile-mediated interspecific plant interaction promotes root colonization by beneficial bacteria via induced shifts in root exudation. Microbiome.

[51] Banerjee S., Schlaeppi K., Van Der Heijden M.G. (2018). Keystone taxa as drivers of microbiome structure and functioning. Nat. Rev. Microbiol..

[52] Kaufmann H., Salvador C., Salazar V.W., Cruz N., Dias G.M., Tschoeke D., Campos L., Sawabe T., Miyazaki M., Maruyama F. (2025). Genomic Repertoire of Twenty-Two Novel Vibrionaceae Species Isolated from Marine Sediments. Microb. Ecol..

