# Supplementary material for: Plant Growth-Promoting Rhizobacteria Enhance Sweet Cherry Root System Development Through the Production of Volatile Organic Compounds
Source: Foods. 2025 Jul 3;14(13):2369. doi: 10.3390/foods14132369 (PMC12249333; doi:10.3390/foods14132369)
Supplement: Supplementary file 1 [file foods-14-02369-s001.zip › foods-3700473-supplementary.pdf]

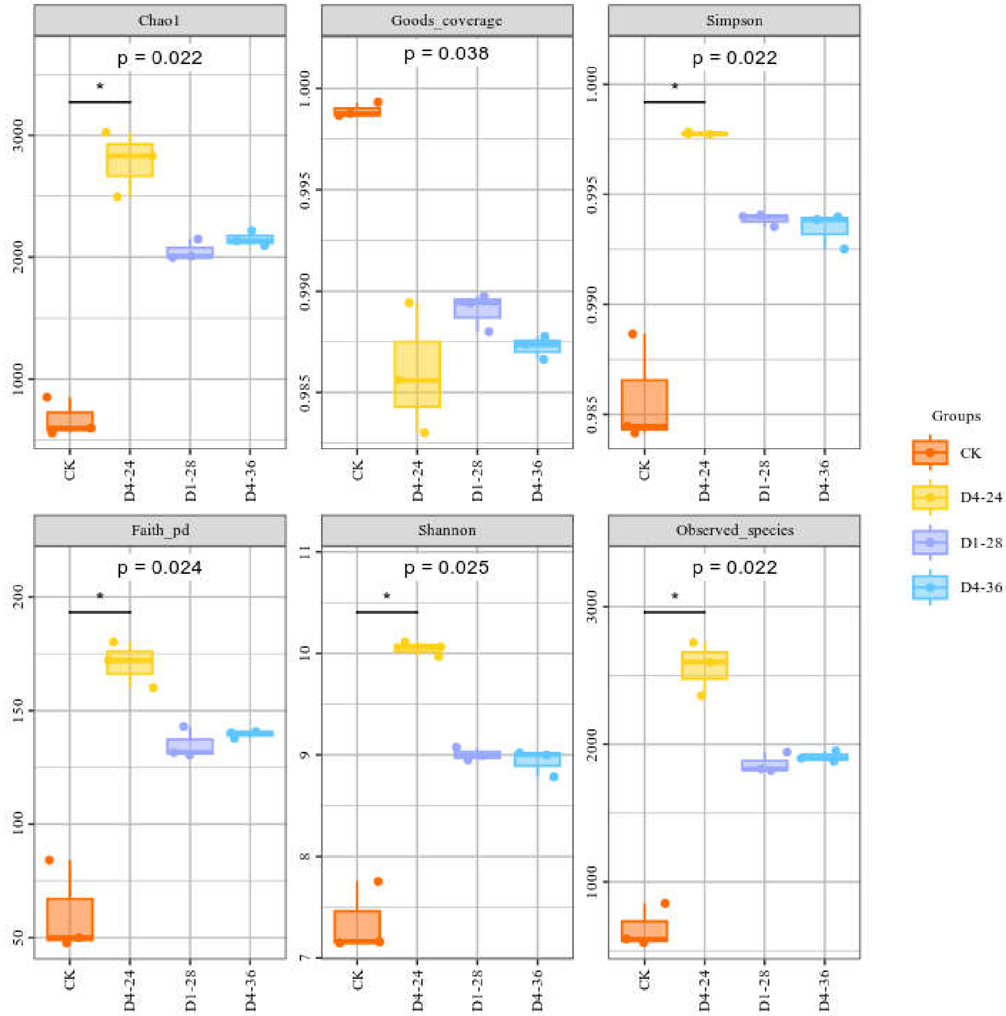

Figure S1. Effect of bacterial VOCs treatment on the bacterial alpha diversity index (Genus level) in cherry roots

Table S1 Primers for quantitative real-time PCR in this study

Gene ID	Gene sequence	Gene length
PavIAA16<br>LOC110754301	F: AGGCTCAAGTTGTGGGTTGG<br>R: CATCTGACCCGCAGTTACCA	249 bp
PavIAA29<br>LOC110763768	F: TGAAGGTGAAGATGGAAGGGGTG<br>R: GATGAAGGTTTGCCAGGGAACA	209 bp/221 bp
PavIAA26<br>LOC110760757	F: CGGTACACGAAATGAGGAGGCA<br>R: CGTGGAAGACATAGGGGCAAAG	232 bp
PavIAA6<br>LOC110760082	F: CCAAGATTGGTGGACAAAAACG<br>R: CCGATAAGAACACACTGGAGGC	150 bp
PavIAA27<br>LOC110751083	F: TCCCCAGAGAGAGATGGTGG<br>R: ACCTCTGGGAGAGAGCAAGT	201 bp
PavIAA21<br>LOC110769500	F: CTGCACAAGCCAAGCAAGAG<br>R: ACATAAAGGCACCCAGACCC	223 bp
PavIAA4<br>LOC110763581	F: GAGGTTCAAGCAGTAGGGCA<br>R: AGGTGCTCCATCCATGCTTAC	246 bp
PavIAA13<br>LOC110756352	F: AAGGCACACTTGGGCTACTC<br>R: GATTCTACCACGCTCACCCC	236 bp
PavIAA33<br>LOC110753474	F: ACGGTGCTAGTCTCCACAAC<br>R: CACATCGCTACCATCCACGA	185 bp
PavARF6<br>LOC110774025	F: ATAATGGGGACCGCGGAATC<br>R: AGGGATGTAGGAAGCGGTCT	181 bp
PavARF9<br>LOC110771748	F: TGGAACCACTGAAGGACGTG<br>R: AGCCATCCCCTGCATTTGAA	187 bp
PavAUX2<br>LOC110753770	F: GGTGTGGGTTTTCGTGGTAGGG<br>R: GGATAGGAGCAGGAGCTGAAGTTG	161 bp
PavPIN2<br>LOC110772015	F: GGCTGGTGTGGCGTATGTAT<br>R: AGGCCATTCAGCCTCAACAA	238 bp
PavPIN3<br>LOC110758039	F: GGAGCAATGGTTGGTTTTGTGA<br>R: CTACAAGAGGCAGGGCAACA	238 bp
PavPIN5<br>LOC110760438	F: GTGCCACTCTACTTTGCCCT<br>R: AAGCACCGCCACGATTATGA	216 bp
PavGH3.1<br>LOC110759585	F: CATAGAGGAGACGACCAGGAACA<br>R: GGAACTCAGAGATGGGATGAGAGCT	239 bp
PavGH3.2<br>LOC110767953	F: CTCCCATCTTGTCAGCTCACCCA<br>R: GCTCACGAACAGAAAGTACAGGCCC	197 bp
PavRSP3<br>LOC110749254	F: TCAAGGTCAGGTAAGGGGGTC<br>R: GTGAGGTGATTGTTAGTGGAAAGC	223 bp
